# 2-(Methyl­sulfan­yl)cyclo­dodeca­none tosyl­hydrazone

**DOI:** 10.1107/S1600536808005515

**Published:** 2008-03-05

**Authors:** Xiao-Jing Yan, Xiao-Mei Liang, Shu-Hui Jin, Dao-Quan Wang

**Affiliations:** a Key Laboratory of Pesticide Chemistry and Application Technology, Department of Applied Chemistry, China Agricultural University, Beijing 100094, People’s Republic of China

## Abstract

The title compound, C_20_H_32_N_2_O_2_S_2_, has been synthesized by the reaction of α-methyl­sulfanylcyclo­dodeca­none and *p*-toluene­sulfonyl­hydrazine. In the crystal structure, the conformation of the non-benzenoid ring is [3333] and the methyl­sulfanyl group is in the α-side *exo* position. The mol­ecules are linked by inter­molecular N—H⋯S hydrogen bonds.

## Related literature

For related literature, see: Li *et al.*(2005 ▶); Lu *et al.* (2004 ▶); Song *et al.* (2005 ▶); Wang *et al.* (2002 ▶, 2007 ▶).
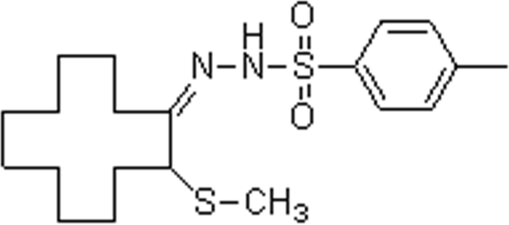


## Experimental

### 

#### Crystal data

C_20_H_32_N_2_O_2_S_2_
*M* *_r_* = 396.60
Monoclinic, 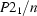

*a* = 8.4374 (7) Å
*b* = 11.5276 (10) Å
*c* = 21.7836 (19) Å
β = 92.530 (2)°
*V* = 2116.7 (3) Å^3^
*Z* = 4
Mo *K*α radiation
μ = 0.27 mm^−1^
*T* = 293 (2) K
0.47 × 0.38 × 0.28 mm

#### Data collection

Bruker SMART CCD area-detector diffractometer
Absorption correction: multi-scan (*SADABS*; Sheldrick, 1996 ▶) *T*_min_ = 0.778, *T*_max_ = 1.000 (expected range = 0.722–0.929)
12199 measured reflections
4615 independent reflections
3650 reflections with *I* > 2σ(*I*)
*R*_int_ = 0.061

#### Refinement

*R*[*F* ^2^ > 2σ(*F* ^2^)] = 0.049
*wR*(*F* ^2^) = 0.131
*S* = 1.03
4615 reflections
241 parameters
1 restraint
H atoms treated by a mixture of independent and constrained refinement
Δρ_max_ = 0.41 e Å^−3^
Δρ_min_ = −0.23 e Å^−3^

### 

Data collection: *SMART* (Bruker, 2000 ▶); cell refinement: *SAINT-Plus* (Bruker, 2000 ▶); data reduction: *SAINT-Plus*; program(s) used to solve structure: *SHELXS97* (Sheldrick, 2008 ▶); program(s) used to refine structure: *SHELXL97* (Sheldrick, 2008 ▶); molecular graphics: *SHELXTL* (Sheldrick, 2008 ▶); software used to prepare material for publication: *SHELXTL*.

## Supplementary Material

Crystal structure: contains datablocks I, global. DOI: 10.1107/S1600536808005515/fl2190sup1.cif

Structure factors: contains datablocks I. DOI: 10.1107/S1600536808005515/fl2190Isup2.hkl

Additional supplementary materials:  crystallographic information; 3D view; checkCIF report

## Figures and Tables

**Table 1:** Hydrogen-bond geometry (Å, °)

*D*—H⋯*A*	*D*—H	H⋯*A*	*D*⋯*A*	*D*—H⋯*A*
N2—H2⋯S1^i^	0.846 (15)	2.786 (15)	3.6223 (18)	170 (2)

Symmetry code: (i) 
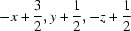
.

**Fig. 1.**
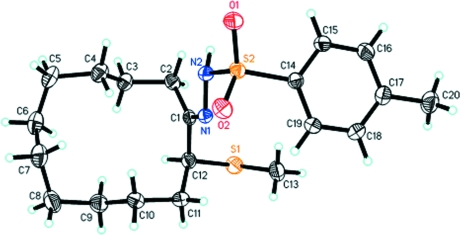
The molecular structure of (I), showing 30% probability displacement ellipsoids and the atom-numbering scheme.

**Fig. 2.**
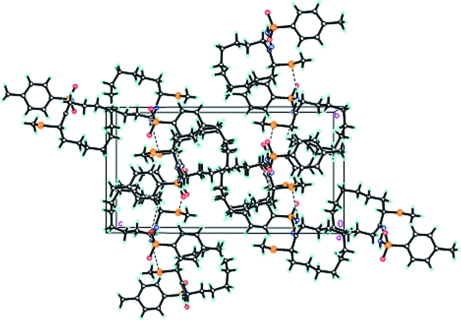
The crystal packing of (I). Intermolecular hydrogen bonds are shown as dashed lines.

## supplementary crystallographic information

### Comment

Many derivatives of cyclododecanone have bioactivity that has attracted much attention from chemists (Song *et al.*, 2005; Li *et al.*, 2005). In order to understand the structure-activity relationships of these materials, it is necessary to study their stereochemistry and overall conformation. We have studied a number of α-monosubstituted cyclododecanones with some fruitful results (Wang *et al.*,2002; Lu *et al.*., 2004). Recently, we found a very interesting conformational phenomenon in the condensation products resulting from on the reactions of α-monosubstituted cyclododecanones with hydroxylamine and thiosemicarbazide (Wang *et al.*, 2007). In these compounds the parent ring has a [3333] conformation and the substituting group is at the α-side-*exo* or α-corner-*anti*position. These results were rationalized by "corner-position carbonyl participation" of raw materials, memory effects and H-bonding between amine derivatives and α-monosubstituted cyclododecanones. To further understand the above results, we synthesized the title compound (I) by the reaction of α-methylsulfanylcyclododecanone and *p*-toluenesulfonylhydrazine. The X-ray analysis further confirmed the validity of our proposed explanation.

The molecular structure of the title compound is given in Fig.1. In the crystal, the parent ring has the [3333] conformation found in the other molecules and the methylsulfanyl group is at α-side-*exo* position. The molecules are linked by intermolecular N—H···S hydrogen bonds (Table 1 and Fig.2).

### Experimental

α-Methylsulfanylcyclododecanone(228 mg, 1.0 mmol) was dissolved in 10 ml absolute ethanol along with *p*-toluenesulfonylhydrazine (279 mg, 1.5 mmol) and a catalytic amount of *p*-toluenesulfonic acid. The reaction mixture was heated to reflux under nitrogen for 5 h and cooled. After removal of the solvent under reduced pressure, the crude product was purified by column chromatography on silica gel (200–300 mesh) using hexane/ethyl acetate (10:1,*v*/*v*) as the eluent, and recrystallized from methanol to give a pure colorless crystal (yield 76%, m.p. 136–138 °C) suitable for X-ray diffraction.

### Refinement

All non-hydrogen atoms were refined with anisotropic displacement parameters. The carbon-bound H atoms were placed at calculated positions, with C—H = 0.93–0.98 Å, and included in the final cycles of refinement using a riding model with *U*_iso _(H) = 1.2 or 1.5(methyl) *U*_eq _(parent atom). The H atoms attached to N2 was located in a difference Fourier map, and was refined with a distance of N–H 0.85 (1) Å.

### Crystal data

**Table:**

C_20_H_32_N_2_O_2_S_2_	*F*_000_ = 856
*M**_r_* = 396.60	*D*_x_ = 1.245 Mg m^−^^3^
Monoclinic, *P*2_1_/*n*	Mo *K*α radiation λ = 0.71073 Å
*a* = 8.4374 (7) Å	Cell parameters from 3644 reflections
*b* = 11.5276 (10) Å	θ = 4.8–55.1º
*c* = 21.7836 (19) Å	µ = 0.27 mm^−^^1^
β = 92.530 (2)º	*T* = 293 (2) K
*V* = 2116.7 (3) Å^3^	Prismatic, colorless
*Z* = 4	0.47 × 0.38 × 0.28 mm

### Data collection

**Table:**

Bruker SMART CCD area-detector diffractometer	4615 independent reflections
Radiation source: fine-focus sealed tube	3650 reflections with *I* > 2σ(*I*)
Monochromator: graphite	*R*_int_ = 0.061
*T* = 293(2) K	θ_max_ = 27.0º
φ and ω scans	θ_min_ = 1.9º
Absorption correction: multi-scan(SADABS; Sheldrick, 1996)	*h* = −10→10
*T*_min_ = 0.778, *T*_max_ = 1.000	*k* = −14→13
12199 measured reflections	*l* = −27→18

### Refinement

**Table:**

Refinement on *F*^2^	Secondary atom site location: difference Fourier map
Least-squares matrix: full	Hydrogen site location: inferred from neighbouring sites
*R*[*F*^2^ > 2σ(*F*^2^)] = 0.049	H atoms treated by a mixture of independent and constrained refinement
*wR*(*F*^2^) = 0.131	*w* = 1/[σ^2^(*F*_o_^2^) + (0.0655*P*)^2^] where *P* = (*F*_o_^2^ + 2*F*_c_^2^)/3
*S* = 1.03	(Δ/σ)_max_ = 0.001
4615 reflections	Δρ_max_ = 0.41 e Å^−^^3^
241 parameters	Δρ_min_ = −0.23 e Å^−^^3^
1 restraint	Extinction correction: none
Primary atom site location: structure-invariant direct methods	

### Special details

**Table:**

Geometry. All e.s.d.'s (except the e.s.d. in the dihedral angle between two l.s. planes) are estimated using the full covariance matrix. The cell e.s.d.'s are taken into account individually in the estimation of e.s.d.'s in distances, angles and torsion angles; correlations between e.s.d.'s in cell parameters are only used when they are defined by crystal symmetry. An approximate (isotropic) treatment of cell e.s.d.'s is used for estimating e.s.d.'s involving l.s. planes.
Refinement. Refinement of *F*^2^ against ALL reflections. The weighted *R*-factor *wR* and goodness of fit *S* are based on *F*^2^, conventional *R*-factors *R* are based on *F*, with *F* set to zero for negative *F*^2^. The threshold expression of *F*^2^ > σ(*F*^2^) is used only for calculating *R*-factors(gt) *etc*. and is not relevant to the choice of reflections for refinement. *R*-factors based on *F*^2^ are statistically about twice as large as those based on *F*, and *R*- factors based on ALL data will be even larger.

### Fractional atomic coordinates and isotropic or equivalent isotropic displacement parameters (Å^2^)

**Table:**

	*x*	*y*	*z*	*U*_iso_*/*U*_eq_	
S1	0.84188 (7)	0.86341 (5)	0.29057 (3)	0.05522 (19)	
S2	0.28829 (5)	1.10533 (4)	0.20128 (2)	0.03749 (15)	
O1	0.24538 (17)	1.21632 (12)	0.17655 (6)	0.0515 (4)	
O2	0.19940 (15)	1.00460 (12)	0.18414 (7)	0.0490 (4)	
N1	0.53858 (18)	0.97987 (13)	0.19883 (7)	0.0360 (4)	
N2	0.47071 (18)	1.08519 (14)	0.17949 (7)	0.0372 (4)	
C1	0.6872 (2)	0.96614 (16)	0.19183 (8)	0.0358 (4)	
C2	0.8013 (2)	1.05110 (18)	0.16582 (9)	0.0427 (5)	
H2A	0.8964	1.0541	0.1925	0.051*	
H2B	0.7536	1.1277	0.1655	0.051*	
C3	0.8478 (2)	1.02089 (19)	0.10087 (9)	0.0471 (5)	
H3A	0.9303	1.0739	0.0889	0.056*	
H3B	0.8918	0.9431	0.1010	0.056*	
C4	0.7115 (3)	1.0267 (2)	0.05364 (10)	0.0529 (6)	
H4A	0.6746	1.1063	0.0507	0.063*	
H4B	0.6249	0.9799	0.0678	0.063*	
C5	0.7525 (3)	0.9853 (2)	−0.01033 (10)	0.0611 (6)	
H5A	0.6654	1.0047	−0.0391	0.073*	
H5B	0.8456	1.0269	−0.0229	0.073*	
C6	0.7844 (3)	0.8560 (2)	−0.01425 (11)	0.0636 (7)	
H6A	0.8664	0.8360	0.0165	0.076*	
H6B	0.8255	0.8393	−0.0542	0.076*	
C7	0.6409 (3)	0.7787 (2)	−0.00514 (11)	0.0666 (7)	
H7A	0.5638	0.8216	0.0175	0.080*	
H7B	0.5921	0.7598	−0.0451	0.080*	
C8	0.6798 (3)	0.6672 (2)	0.02873 (12)	0.0710 (7)	
H8A	0.7644	0.6280	0.0083	0.085*	
H8B	0.5874	0.6172	0.0262	0.085*	
C9	0.7308 (3)	0.6842 (2)	0.09646 (11)	0.0596 (6)	
H9A	0.7750	0.6119	0.1123	0.072*	
H9B	0.8141	0.7422	0.0993	0.072*	
C10	0.5981 (3)	0.72134 (18)	0.13636 (10)	0.0491 (5)	
H10A	0.5183	0.6607	0.1355	0.059*	
H10B	0.5491	0.7904	0.1186	0.059*	
C11	0.6475 (3)	0.74684 (18)	0.20316 (10)	0.0495 (5)	
H11A	0.5527	0.7598	0.2259	0.059*	
H11B	0.7006	0.6790	0.2206	0.059*	
C12	0.7559 (2)	0.85050 (17)	0.21215 (9)	0.0434 (5)	
H12	0.8455	0.8364	0.1860	0.052*	
C13	0.6686 (3)	0.8781 (2)	0.33481 (11)	0.0699 (7)	
H13A	0.6090	0.9447	0.3208	0.105*	
H13B	0.7000	0.8877	0.3774	0.105*	
H13C	0.6041	0.8099	0.3299	0.105*	
C14	0.2972 (2)	1.11577 (16)	0.28203 (9)	0.0368 (4)	
C15	0.3174 (2)	1.22352 (17)	0.30923 (9)	0.0429 (5)	
H15	0.3252	1.2896	0.2851	0.051*	
C16	0.3258 (2)	1.23198 (18)	0.37211 (10)	0.0485 (5)	
H16	0.3384	1.3045	0.3903	0.058*	
C17	0.3159 (3)	1.13478 (19)	0.40892 (10)	0.0481 (5)	
C18	0.2959 (3)	1.02835 (18)	0.38062 (10)	0.0530 (6)	
H18	0.2896	0.9622	0.4048	0.064*	
C19	0.2849 (3)	1.01732 (17)	0.31766 (10)	0.0475 (5)	
H19	0.2695	0.9450	0.2995	0.057*	
C20	0.3224 (4)	1.1469 (2)	0.47810 (11)	0.0711 (7)	
H20A	0.3608	1.2228	0.4893	0.107*	
H20B	0.3924	1.0892	0.4959	0.107*	
H20C	0.2180	1.1365	0.4931	0.107*	
H2	0.522 (2)	1.1482 (14)	0.1827 (10)	0.049 (6)*	

### Atomic displacement parameters (Å^2^)

**Table:**

	*U*^11^	*U*^22^	*U*^33^	*U*^12^	*U*^13^	*U*^23^
S1	0.0623 (4)	0.0541 (4)	0.0480 (3)	0.0090 (3)	−0.0123 (3)	0.0002 (3)
S2	0.0374 (3)	0.0370 (3)	0.0381 (3)	0.00631 (19)	0.0012 (2)	−0.0006 (2)
O1	0.0597 (9)	0.0459 (8)	0.0486 (9)	0.0192 (7)	−0.0012 (7)	0.0047 (7)
O2	0.0430 (8)	0.0507 (8)	0.0529 (9)	−0.0030 (6)	−0.0030 (7)	−0.0071 (7)
N1	0.0418 (9)	0.0335 (8)	0.0328 (8)	0.0047 (7)	0.0035 (7)	0.0019 (6)
N2	0.0411 (9)	0.0334 (9)	0.0376 (9)	0.0032 (7)	0.0054 (7)	0.0024 (7)
C1	0.0411 (10)	0.0376 (10)	0.0287 (9)	0.0050 (8)	0.0016 (8)	−0.0046 (8)
C2	0.0384 (10)	0.0427 (11)	0.0473 (12)	−0.0002 (8)	0.0047 (9)	−0.0063 (9)
C3	0.0393 (10)	0.0561 (13)	0.0464 (12)	0.0003 (9)	0.0094 (9)	0.0025 (10)
C4	0.0492 (12)	0.0631 (15)	0.0466 (13)	0.0093 (11)	0.0049 (10)	0.0085 (11)
C5	0.0593 (14)	0.0856 (18)	0.0387 (12)	0.0064 (13)	0.0036 (11)	0.0137 (12)
C6	0.0608 (15)	0.0898 (19)	0.0408 (13)	0.0071 (13)	0.0102 (11)	−0.0017 (12)
C7	0.0725 (16)	0.0837 (18)	0.0426 (13)	0.0016 (14)	−0.0079 (12)	−0.0062 (13)
C8	0.0862 (19)	0.0698 (17)	0.0574 (16)	0.0073 (14)	0.0065 (14)	−0.0220 (13)
C9	0.0712 (15)	0.0528 (14)	0.0546 (14)	0.0144 (12)	0.0005 (12)	−0.0008 (11)
C10	0.0562 (13)	0.0407 (11)	0.0504 (13)	−0.0028 (10)	0.0011 (10)	−0.0023 (10)
C11	0.0623 (13)	0.0387 (11)	0.0476 (13)	0.0007 (10)	0.0046 (11)	0.0055 (9)
C12	0.0467 (11)	0.0448 (12)	0.0386 (11)	0.0081 (9)	0.0012 (9)	−0.0022 (9)
C13	0.099 (2)	0.0649 (16)	0.0459 (14)	0.0174 (14)	0.0083 (14)	−0.0074 (12)
C14	0.0350 (9)	0.0365 (10)	0.0395 (11)	0.0030 (8)	0.0065 (8)	0.0005 (8)
C15	0.0470 (11)	0.0355 (10)	0.0466 (12)	−0.0006 (9)	0.0078 (9)	0.0014 (9)
C16	0.0549 (12)	0.0418 (11)	0.0491 (13)	−0.0045 (10)	0.0067 (10)	−0.0073 (10)
C17	0.0495 (12)	0.0550 (13)	0.0402 (12)	−0.0020 (10)	0.0069 (10)	−0.0008 (10)
C18	0.0706 (14)	0.0430 (12)	0.0462 (13)	0.0004 (11)	0.0127 (11)	0.0095 (10)
C19	0.0621 (13)	0.0336 (10)	0.0476 (12)	0.0014 (9)	0.0116 (10)	−0.0019 (9)
C20	0.093 (2)	0.0784 (18)	0.0421 (13)	−0.0100 (15)	0.0074 (14)	−0.0047 (12)

### Geometric parameters (Å, °)

**Table:**

S1—C13	1.794 (3)	C8—H8A	0.9700
S1—C12	1.832 (2)	C8—H8B	0.9700
S2—O2	1.4232 (14)	C9—C10	1.509 (3)
S2—O1	1.4287 (14)	C9—H9A	0.9700
S2—N2	1.6470 (16)	C9—H9B	0.9700
S2—C14	1.761 (2)	C10—C11	1.524 (3)
N1—C1	1.280 (2)	C10—H10A	0.9700
N1—N2	1.399 (2)	C10—H10B	0.9700
N2—H2	0.846 (15)	C11—C12	1.512 (3)
C1—C2	1.501 (3)	C11—H11A	0.9700
C1—C12	1.512 (3)	C11—H11B	0.9700
C2—C3	1.525 (3)	C12—H12	0.9800
C2—H2A	0.9700	C13—H13A	0.9600
C2—H2B	0.9700	C13—H13B	0.9600
C3—C4	1.510 (3)	C13—H13C	0.9600
C3—H3A	0.9700	C14—C19	1.382 (3)
C3—H3B	0.9700	C14—C15	1.383 (3)
C4—C5	1.527 (3)	C15—C16	1.372 (3)
C4—H4A	0.9700	C15—H15	0.9300
C4—H4B	0.9700	C16—C17	1.383 (3)
C5—C6	1.517 (3)	C16—H16	0.9300
C5—H5A	0.9700	C17—C18	1.380 (3)
C5—H5B	0.9700	C17—C20	1.512 (3)
C6—C7	1.524 (3)	C18—C19	1.376 (3)
C6—H6A	0.9700	C18—H18	0.9300
C6—H6B	0.9700	C19—H19	0.9300
C7—C8	1.510 (3)	C20—H20A	0.9600
C7—H7A	0.9700	C20—H20B	0.9600
C7—H7B	0.9700	C20—H20C	0.9600
C8—C9	1.531 (3)		
			
C13—S1—C12	102.13 (11)	H8A—C8—H8B	107.6
O2—S2—O1	120.65 (9)	C10—C9—C8	114.0 (2)
O2—S2—N2	107.32 (8)	C10—C9—H9A	108.7
O1—S2—N2	104.01 (9)	C8—C9—H9A	108.7
O2—S2—C14	108.44 (9)	C10—C9—H9B	108.7
O1—S2—C14	108.32 (8)	C8—C9—H9B	108.7
N2—S2—C14	107.36 (9)	H9A—C9—H9B	107.6
C1—N1—N2	117.49 (15)	C9—C10—C11	115.22 (18)
N1—N2—S2	114.25 (12)	C9—C10—H10A	108.5
N1—N2—H2	121.3 (14)	C11—C10—H10A	108.5
S2—N2—H2	109.5 (15)	C9—C10—H10B	108.5
N1—C1—C2	127.74 (17)	C11—C10—H10B	108.5
N1—C1—C12	116.03 (17)	H10A—C10—H10B	107.5
C2—C1—C12	116.23 (16)	C12—C11—C10	114.46 (17)
C1—C2—C3	113.37 (16)	C12—C11—H11A	108.6
C1—C2—H2A	108.9	C10—C11—H11A	108.6
C3—C2—H2A	108.9	C12—C11—H11B	108.6
C1—C2—H2B	108.9	C10—C11—H11B	108.6
C3—C2—H2B	108.9	H11A—C11—H11B	107.6
H2A—C2—H2B	107.7	C1—C12—C11	115.91 (17)
C4—C3—C2	113.72 (17)	C1—C12—S1	109.40 (13)
C4—C3—H3A	108.8	C11—C12—S1	113.40 (15)
C2—C3—H3A	108.8	C1—C12—H12	105.8
C4—C3—H3B	108.8	C11—C12—H12	105.8
C2—C3—H3B	108.8	S1—C12—H12	105.8
H3A—C3—H3B	107.7	S1—C13—H13A	109.5
C3—C4—C5	114.30 (18)	S1—C13—H13B	109.5
C3—C4—H4A	108.7	H13A—C13—H13B	109.5
C5—C4—H4A	108.7	S1—C13—H13C	109.5
C3—C4—H4B	108.7	H13A—C13—H13C	109.5
C5—C4—H4B	108.7	H13B—C13—H13C	109.5
H4A—C4—H4B	107.6	C19—C14—C15	120.52 (19)
C6—C5—C4	114.00 (19)	C19—C14—S2	120.27 (15)
C6—C5—H5A	108.8	C15—C14—S2	119.21 (15)
C4—C5—H5A	108.8	C16—C15—C14	119.43 (18)
C6—C5—H5B	108.8	C16—C15—H15	120.3
C4—C5—H5B	108.8	C14—C15—H15	120.3
H5A—C5—H5B	107.6	C15—C16—C17	121.32 (19)
C5—C6—C7	115.0 (2)	C15—C16—H16	119.3
C5—C6—H6A	108.5	C17—C16—H16	119.3
C7—C6—H6A	108.5	C18—C17—C16	118.1 (2)
C5—C6—H6B	108.5	C18—C17—C20	121.7 (2)
C7—C6—H6B	108.5	C16—C17—C20	120.2 (2)
H6A—C6—H6B	107.5	C19—C18—C17	121.93 (19)
C8—C7—C6	113.8 (2)	C19—C18—H18	119.0
C8—C7—H7A	108.8	C17—C18—H18	119.0
C6—C7—H7A	108.8	C18—C19—C14	118.72 (19)
C8—C7—H7B	108.8	C18—C19—H19	120.6
C6—C7—H7B	108.8	C14—C19—H19	120.6
H7A—C7—H7B	107.7	C17—C20—H20A	109.5
C7—C8—C9	114.1 (2)	C17—C20—H20B	109.5
C7—C8—H8A	108.7	H20A—C20—H20B	109.5
C9—C8—H8A	108.7	C17—C20—H20C	109.5
C7—C8—H8B	108.7	H20A—C20—H20C	109.5
C9—C8—H8B	108.7	H20B—C20—H20C	109.5
			
C1—N1—N2—S2	−169.49 (13)	C10—C11—C12—C1	62.7 (2)
O2—S2—N2—N1	−51.04 (15)	C10—C11—C12—S1	−169.51 (15)
O1—S2—N2—N1	−179.96 (12)	C13—S1—C12—C1	70.58 (16)
C14—S2—N2—N1	65.37 (14)	C13—S1—C12—C11	−60.51 (17)
N2—N1—C1—C2	1.0 (3)	O2—S2—C14—C19	26.37 (19)
N2—N1—C1—C12	−178.92 (15)	O1—S2—C14—C19	158.95 (16)
N1—C1—C2—C3	−105.1 (2)	N2—S2—C14—C19	−89.30 (18)
C12—C1—C2—C3	74.8 (2)	O2—S2—C14—C15	−153.98 (15)
C1—C2—C3—C4	65.0 (2)	O1—S2—C14—C15	−21.41 (18)
C2—C3—C4—C5	−174.27 (19)	N2—S2—C14—C15	90.35 (16)
C3—C4—C5—C6	68.5 (3)	C19—C14—C15—C16	0.2 (3)
C4—C5—C6—C7	67.2 (3)	S2—C14—C15—C16	−179.41 (15)
C5—C6—C7—C8	−144.2 (2)	C14—C15—C16—C17	0.6 (3)
C6—C7—C8—C9	68.6 (3)	C15—C16—C17—C18	−0.5 (3)
C7—C8—C9—C10	70.6 (3)	C15—C16—C17—C20	−178.8 (2)
C8—C9—C10—C11	−176.03 (19)	C16—C17—C18—C19	−0.3 (3)
C9—C10—C11—C12	65.8 (2)	C20—C17—C18—C19	177.9 (2)
N1—C1—C12—C11	34.1 (2)	C17—C18—C19—C14	1.2 (3)
C2—C1—C12—C11	−145.82 (18)	C15—C14—C19—C18	−1.1 (3)
N1—C1—C12—S1	−95.61 (17)	S2—C14—C19—C18	178.55 (16)
C2—C1—C12—S1	84.45 (18)		

### Hydrogen-bond geometry (Å, °)

**Table:**

*D*—H···*A*	*D*—H	H···*A*	*D*···*A*	*D*—H···*A*
N2—H2···S1^i^	0.846 (15)	2.786 (15)	3.6223 (18)	170 (2)

Symmetry codes: (i) −*x*+3/2, *y*+1/2, −*z*+1/2.
